# Imaging findings of high-grade penetrating renal trauma in a pediatric patient

**DOI:** 10.1016/j.radcr.2025.09.005

**Published:** 2025-09-29

**Authors:** Kawtar El Jebbouri, Ihssane Laasri, Fatima Chait, Siham El Haddad, Nazik Allali, Latifa Chat

**Affiliations:** Department of Radiology, Children’s Hospital, Mohamed V University, Rabat, Morocco

**Keywords:** Renal trauma, Penetrating injuries, Pediatric trauma

## Abstract

Renal trauma is a significant cause of morbidity in pediatric patients, with penetrating injuries representing a smaller but severe subset. We present the case of an 11-year-old boy with a penetrating stab wound to the left flank causing hemodynamic instability. Initial ultrasound revealed free fluid in the perirenal space, prompting a contrast-enhanced thoracoabdominal-pelvic CT scan, which demonstrated a grade V left mid-renal laceration extending to the hilum with devascularization of the medial renal parenchyma and active arterial contrast extravasation near the renal pedicle. A large perirenal and pararenal hematoma extended into the intraperitoneal space, confirming ongoing hemorrhage. Imaging findings guided a multidisciplinary decision for conservative management under intensive monitoring. This case underscores the critical role of multiphase CT imaging in accurately grading pediatric penetrating renal trauma, identifying vascular injury and hemorrhage, and guiding appropriate management decisions.

## Introduction

Traumatic injuries are the leading cause of death in children, with the kidneys being the most frequently injured organ in the genitourinary system, involved in up to 3.25% of trauma cases. Penetrating trauma, primarily due to gunshot wounds or stabbings, represents a smaller proportion of these cases while the majority of renal injuries in children are caused by blunt trauma [1]. Accurate and timely imaging evaluation, particularly with contrast-enhanced computed tomography (CT), is essential for grading the extent of injury, detecting vascular involvement, and guiding clinical management.

## Case report

An 11-year-old previously healthy teenager was admitted to the emergency department after sustaining a stab wound to the left flank during a physical assault. On arrival, the patient was hemodynamically unstable, with a blood pressure of 72/40 mmHg and a heart rate of 140 bpm. Physical examination revealed a penetrating wound in the left lumbar region, associated with abdominal tenderness and flank ecchymosis. Initial laboratory tests showed a drop in hemoglobin and elevated lactate levels, consistent with hemorrhagic shock.

Resuscitation was initiated immediately with intravenous fluids and blood transfusions, resulting in transient stabilization. A focused assessment with sonography for trauma (FAST) revealed free fluid in the left upper quadrant and perirenal space.

**Fig. 1:**
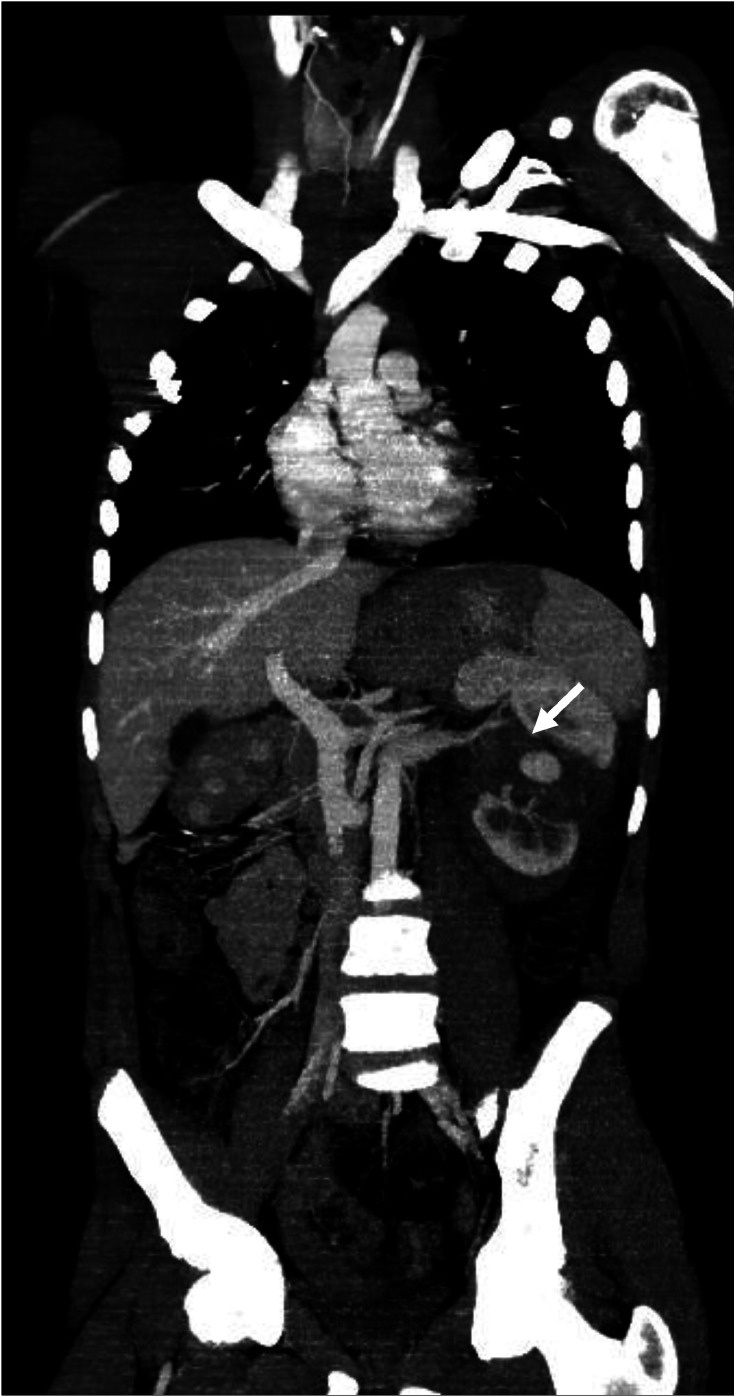
Coronal contrast-enhanced abdominal CT showing a renal laceration extending through the renal hilum with devascularization of the medial portion of the left kidney, involving the region from the lower to mid pole. The left renal artery is patent up to its division; beyond that point, it becomes difficult to delineate.

**Fig. 2:**
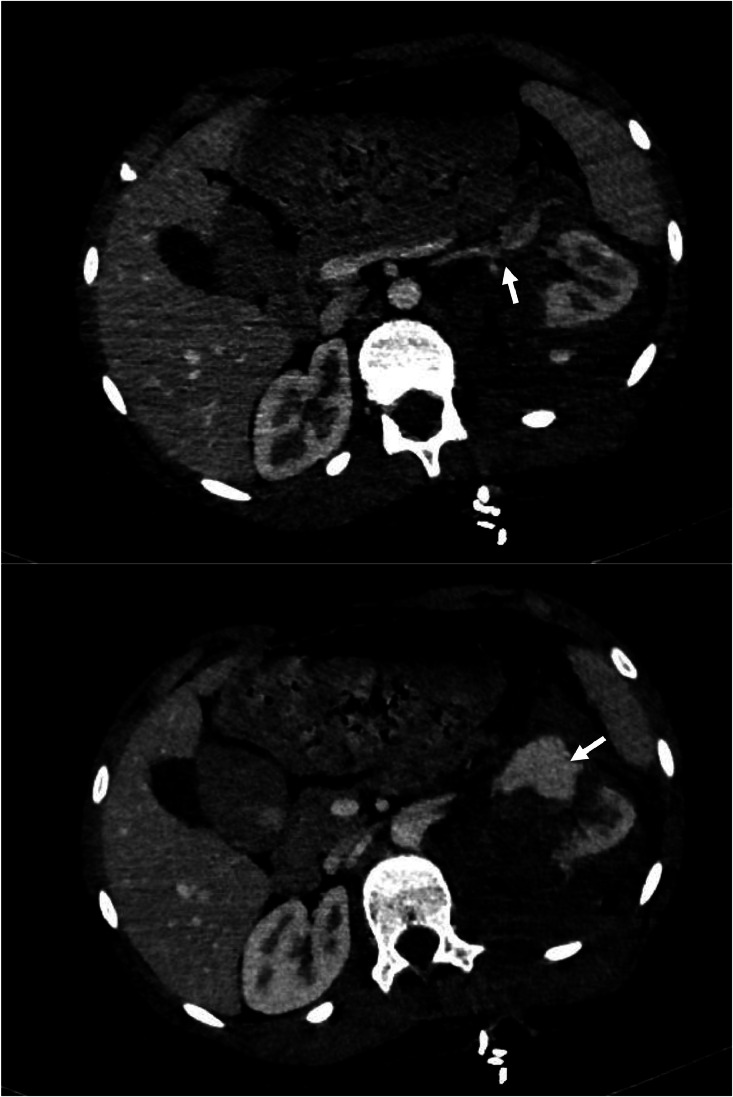
Axial contrast-enhanced abdominal CT showing rupture of the left renal vein (A) with active contrast extravasation (B).

**Fig. 3:**
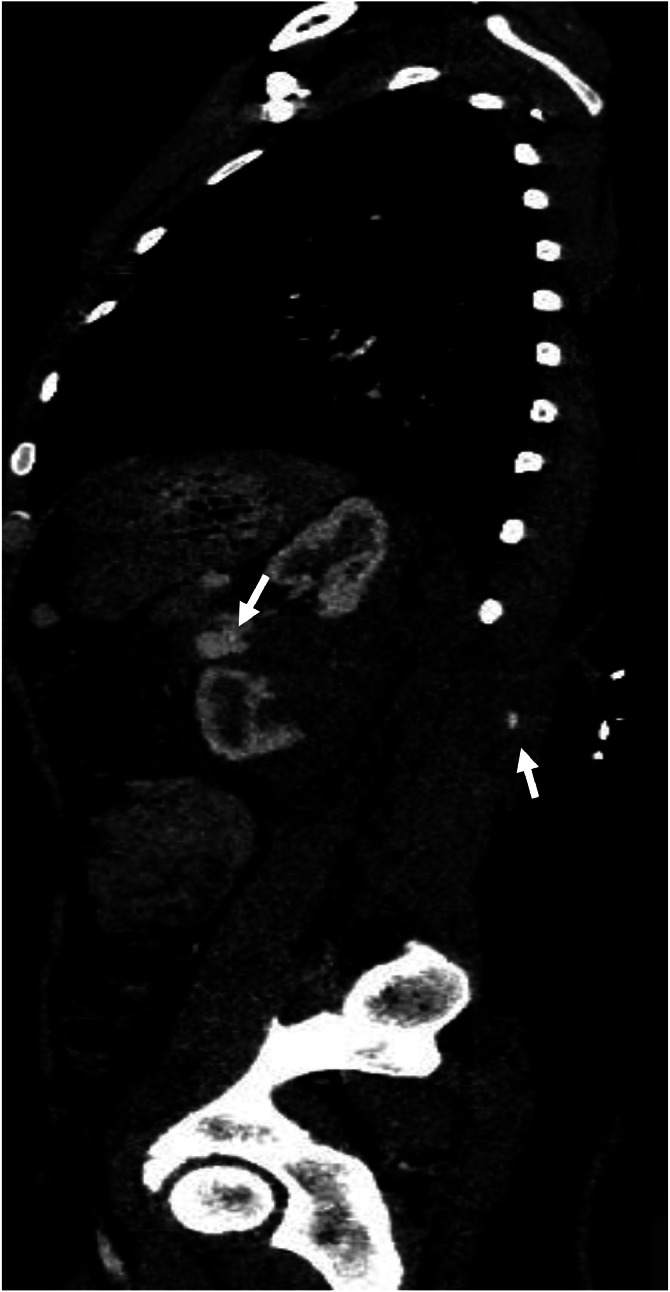
Sagittal contrast-enhanced abdominal CT showing a penetrating wound in the left lumbar region with an entry point adjacent to the L1 vertebra. The wound tract extends through the left longissimus thoracis muscle, associated with emphysematous bubbles and contrast extravasation along the trajectory.

**Fig. 4:**
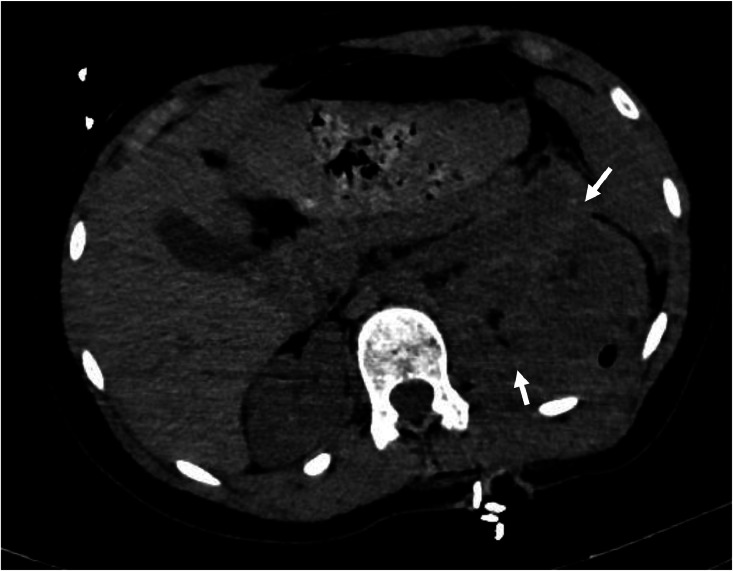
Axial non-contrast abdominal CT showing a large perirenal and pararenal hematoma extending into the intraperitoneal space, consistent with active bleeding.

Once stabilized, a contrast-enhanced thoracoabdominal-pelvic CT (CT TAP) was performed. Imaging revealed a left mid-renal laceration consistent with a *grade V renal injury* according to the American Association for the Surgery of Trauma (AAST). The fracture extended through the renal hilum with *devascularization of the medial portion of the kidney*, involving the region from the lower to mid pole (Fig. 1). There was *extensive contrast extravasation adjacent to the renal pedicle*, indicating active arterial bleeding (Figs. 2 and 3). Additionally, a large *perirenal and pararenal hematoma* was observed, extending into the *intraperitoneal space*, further supporting ongoing hemorrhage (Fig. 4).

Given the extent of the injury and evidence of active bleeding, the patient was admitted to the pediatric intensive care unit for close monitoring and supportive management. Multidisciplinary consultation with pediatric surgery, urology, and interventional radiology was obtained. Despite the severity of the injury, a non-operative approach was initially favored due to hemodynamic stability under close surveillance.

The patient's condition remained stable over the following days, with no further need for transfusions. Follow-up imaging confirmed the stabilization of the hematoma and absence of ongoing bleeding. The patient was discharged on hospital day 10 with preserved renal function and scheduled for outpatient follow-up.

## Discussion

The kidney, despite its retroperitoneal protection, is the most commonly injured genitourinary organ during trauma. While renal injuries can be isolated, 90% occur alongside other injuries. These injuries primarily affect younger men, with an even lower average age in cases of penetrating trauma [1]. Children and adolescents are more vulnerable to kidney trauma due to the larger size of their kidneys relative to the abdominal cavity, weaker connective tissues, less developed musculature, and less ossified ribs. The pediatric kidney’s increased mobility and lower position in the abdomen further heighten its risk. Trauma energy is distributed over a smaller body surface area in children, resulting in greater impact force. Additionally, the close proximity of intra-abdominal organs increases the likelihood of multiple organ injuries. Children are particularly prone to flexion-extension injuries, such as those from pedestrian-vehicle accidents [2].

Blunt trauma is the primary cause of renal injuries, while penetrating trauma, typically caused by firearms and stab wounds, constitutes 10% of cases.

The mechanism of injury in penetrating trauma involves direct damage to the renal parenchyma, excretory system, vascular structures, or even disruption of the peritoneum. Penetrating injuries are frequently linked to nonsterile conditions, increasing the risk of bacterial proliferation within the hematoma or urine leakage. These complications may necessitate surgical debridement or, in severe cases, nephrectomy [3].

Penetrating trauma is classified by projectile velocity: high-velocity (e.g., rifles), medium-velocity (e.g., handguns), and low-velocity (e.g., knife stabs). High-velocity injuries cause widespread tissue damage due to energy transfer, creating shear forces and cavitation beyond the projectile’s path. Lower-velocity injuries are typically confined to the projectile's track [1]. Stab wound location influences outcomes, with anterior abdominal wounds potentially damaging vital renal structures, while posterior wounds primarily affect the renal parenchyma as was the case with our patient, who presented with a penetrating wound in the left lumbar region [4].

The American Association for the Surgery of Trauma (AAST) classification is the most commonly used system for renal trauma, grading injuries from 1 (least severe) to 5 (most severe).

The AAST grading system and its modifications were initially validated for adult trauma cases but continue to be used to classify pediatric injuries [5].

Validated by multiple studies, the AAST grade, along with overall injury severity and the need for transfusion, predicts the likelihood of nephrectomy, morbidity, and mortality. Higher AAST grades are strongly associated with increased risks of surgery and nephrectomy, with gunshot injuries typically resulting in higher grades than blunt trauma [1].

In cases of penetrating trauma, the anatomical locations of entry and exit wounds can provide valuable information regarding the potential for renal involvement [6].

In penetrating trauma, nearly all patients with renal gunshot wounds and up to 60% of those with renal stab wounds sustain concomitant injuries to adjacent organs. In contrast, renal vascular injuries are uncommon following blunt trauma [7]. Penetrating trauma is associated with a significantly higher incidence of renal vascular injury, with reported rates ranging from 15% to 33% [8]. Clinical signs suggestive of potential renal injury include hematuria, a penetrating wound near the renal area, flank bruising or ecchymosis, fractures of the 10th to 12th ribs, the presence of an abdominal mass, and abdominal tenderness or distension. Once significant renal trauma is identified, close clinical monitoring is essential [7].

Contrast-enhanced CT is the gold standard for assessing renal injuries, providing detailed images of renal function, vessels, and associated trauma. USG is commonly used as an initial screening tool due to its portability and ease of use, but it is less sensitive for parenchymal injuries and retroperitoneal hemorrhage. FAST scans help detect hemoperitoneum and guide immediate management decisions. MRI is used in select cases, such as contraindications to contrast, but has limitations due to time and motion artifacts. Angiography is now primarily therapeutic, used for embolization or revascularization. Radiological evaluation follows European Association of Urology (EAU) guidelines [9].

Imaging for pediatric renal trauma follows the ALARA principle to minimize radiation risks [2].

While the European Association of Urology and the European Society of Pediatric Radiology recommend imaging for any level of nonvisible hematuria, the Renal Trauma Subcommittee of the Societé Internationale d'Urologie states that imaging is only required if there are 50 RBCs/HPF. However, for awake, alert children with minimal symptoms or clinical signs of renal injury and microhematuria less than 50 RBCs/HPF, observation without CT scans may be appropriate, using only ultrasonography for screening [7].

CT imaging for renal trauma should include four distinct phases: precontrast, postcontrast arterial (35 seconds after intravenous injection), postcontrast nephrogenic/portal venous (75 seconds after intravenous injection), and delayed (5-10 minutes after intravenous injection). The precontrast phase can detect renal calculi, which influence management, as well as active bleeding or intraparenchymal hematomas. The postcontrast phases are essential for identifying parenchymal and vascular injuries, including active contrast extravasation, damage to other solid organs (such as the liver and pancreas), and physiological variants that may impact treatment decisions. The delayed phase provides detailed visualization of the collecting system and potential ureteric injury. If the delayed phase cannot be performed during the initial assessment due to urgent priorities, it should be carried out as soon as feasible [1].

Follow-up imaging may be necessary to monitor the resolution of injuries. It is generally not required for Grade I-III blunt renal injuries or for Grade IV renal injuries without urinary extravasation. However, follow-up imaging (such as CECT) is recommended for patients with Grade IV injuries showing urinary leakage on prior scans, Grade V renal injuries that were managed conservatively, those displaying signs of complications (such as fever, a drop in hematocrit levels, or clinical instability), and patients with other associated comorbidities [9].

The European Society of Pediatric Radiology, however, advises obtaining routine re-imaging within a slightly shorter interval of 4-24 hours [7].

Improvements in trauma care, CT imaging, and interventional radiology have led to a shift toward conservative management for renal injuries, decreasing the need for surgical intervention. The goal of this approach is to reduce unnecessary surgeries, maintain kidney function, and prevent long-term issues such as the need for dialysis. Most renal injuries are classified as grade I-III (85%), and these can typically be managed without surgery. High-grade injuries (IV and V) can also be treated conservatively in stable patients. In the past, penetrating renal injuries often necessitated immediate nephrectomy, but now some can be managed nonoperatively. Notably, pediatric renal injuries treated at adult hospitals result in nephrectomy rates that are three times higher compared to those treated at pediatric hospitals [2].

In the last 20 years, the approach to managing pediatric renal injuries has shifted primarily towards conservative treatment. Immediate surgical intervention is required for hemodynamically unstable children or those with severe penetrating abdominal injuries. For stable children, a detailed medical history and assessment are crucial. Surgical or interventional radiology management is indicated for expanding or pulsatile renal hematomas, and hemodynamic instability unresponsive to transfusions. Relative indications for surgery include massive urinary extravasation, significant nonviable tissue, arterial injury, and incomplete staging of the injury [7].

Renal injuries can lead to early complications such as urinoma, bleeding, infection, and hypertension, occurring within a month. Late complications may include hydronephrosis, arteriovenous fistula, pyelonephritis, and stone formation. Low-grade injuries typically heal completely, while high-grade injuries often result in permanent scarring, potentially causing urinary obstruction and increasing the risk of stones and infections. Chronic hypertension is uncommon [2].

## Conclusion

Children are more susceptible to renal injury from blunt or penetrating trauma because of their anatomical differences. The preferred method for initial imaging is four-phase computed tomography with intravenous contrast (noncontrast, arterial, nephrographic, and pyelographic phases), although ultrasonography may also be used for children with minimal symptoms. The approach to managing pediatric renal injuries has primarily transitioned to conservative treatment [7].

## Patient consent

Written informed consent for the publication of this case report was obtained from the patient.
